# The protein architecture and allosteric landscape of HNF4α

**DOI:** 10.3389/fendo.2023.1219092

**Published:** 2023-09-04

**Authors:** Fraydoon Rastinejad

**Affiliations:** Nuffield Department of Medicine, Target Discovery Institute (NDMRB), University of Oxford, Oxford, United Kingdom

**Keywords:** HNF4α, MODY, allosteric activation/regulation, nuclear receptor (NR), NR2A1, structural biology

## Abstract

Hepatocyte nuclear factor 4 alpha (HNF4α) is a multi-faceted nuclear receptor responsible for governing the development and proper functioning of liver and pancreatic islet cells. Its transcriptional functions encompass the regulation of vital metabolic processes including cholesterol and fatty acid metabolism, and glucose sensing and control. Various genetic mutations and alterations in HNF4α are associated with diabetes, metabolic disorders, and cancers. From a structural perspective, HNF4α is one of the most comprehensively understood nuclear receptors due to its crystallographically observed architecture revealing interconnected DNA binding domains (DBDs) and ligand binding domains (LBDs). This review discusses key properties of HNF4α, including its mode of homodimerization, its binding to fatty acid ligands, the importance of post-translational modifications, and the mechanistic basis for allosteric functions. The surfaces linking HNF4α’s DBDs and LBDs create a convergence zone that allows signals originating from any one domain to influence distant domains. The HNF4α-DNA complex serves as a prime illustration of how nuclear receptors utilize individual domains for specific functions, while also integrating these domains to create cohesive higher-order architectures that allow signal responsive functions.

## Introduction

The hepatocyte nuclear factor 4 alpha (HNF4α, NR2A1) is a transcription factor first cloned by Frances Sladek in James Darnell’s laboratory and demonstrated to be enriched in liver extracts and identified as a member of the nuclear receptor (NR) family (1). HNF4α is most highly expressed in the liver, pancreatic islets cells, kidney and several other tissues in humans and rodents (2). In the liver and pancreatic islet β-cells, it is a prominent transcriptional regulator that regulates thousands of genes that impact development and physiology (3–5).

In the liver, HNF4α has been identified as a core transcription factor in super-enhancer-associated networks (6, 7). It regulates genetic programs underlying the morphological and functional differentiation of hepatocytes (8). Further roles include regulating genes responsible for cholesterol, drug, fatty acid, and amino acid metabolism, and genes involved in gluconeogenesis and glycolysis (9). Animals lacking HNF4α have high lipid accumulation in liver and show an impairment of liver gluconeogenesis during fasting. Loss of HNF4α reduces the secretion of serum bile acid, cholesterol, and triglycerides (TGs) levels, most likely because of defects in lipid transport and metabolism (10). In non-alcoholic steatohepatitis and high-fat diet-fed diabetic models, liver expression of HNF4-α is significantly reduced (11). In rats with advanced cirrhosis, reduction in HNF4α expression correlates with worsening of liver function (12).

In pancreatic β-cells, HNF4α has roles in development and regeneration (13). In β -cells, HNF4α expression levels rise during fasting and decrease with food intake (14, 15). Hetero- or homozygous deletion of HNF4α from mouse β -cells impairs glucose sensitivity and insulin secretion, eventually causing hyperinsulinemia and glucose intolerance (16). HNF4α is known to cooperate with other types of transcription factors including HNF1α, HNF6 and HNF3α/FOXA2 to effect glucose sensing and control in pancreatic islets (3, 17).

A large number of human genetic variations to HNF4α occur that alter the protein structure and function of HNF4α and are linked with diabetes, metabolic disorders, and some types of cancers. One type of well-studied disease linked to HNF4α is maturity-onset diabetes of the young (MODY). MODY is a rare form of diabetes that is typically caused by mutations in genes involved in insulin secretion and glucose metabolism. HNF4α mutations are the most common cause of MODY type 1 (MODY1), accounting for approximately 30% of cases (18–21). HNF4α MODY1 mutations manifest impaired insulin secretion and reduced sensitivity to insulin.

The HNF4α protein has been suggested as a prominent drug target in metabolic disorders and cancers. Hyperinsulinemic hypoglycemia (HH) is a condition characterized by impaired secretion of insulin in relation to the blood glucose concentration. Heterozygous mutations in HNF4α are associated with transient HH in humans and risk of fetal macrosomia (22, 23). Furthermore, HNF4α can be viewed as a prominent drug target in colon and gastric cancers (24–26).

Due to their small-molecule ligand binding capabilities, NRs have been well exploited as clinical drug targets (27–29). The clear disease involvements shown for HNF4α suggest that modulating its expression levels or its protein function by drug-like molecules could be promising for control of metabolic disorders or cancer. Yet its excessively large repertoire of target genes, particularly in the liver, would suggest that drugs targeting this nuclear receptor would almost certainly produce unwanted pleiotropic effects.

Here I review the key structural properties of the HNF4α protein, pointing to the distinct features of the polypeptide structure that account for its diverse functions as well as sites that produce allosteric sensing and signal propagation. Although HNF4α is a member of the NR family and shares common features with the remaining 47 members, it exhibits unique features in quaternary architecture and ligand-binding abilities. With the structural visualization of its homodimeric complex on its response element DNA, the HNF4α complex has shown itself to be a highly tuned allosteric system. This review focuses on three-dimensional structural information derived from crystallographic analysis, to point out and interpret the functional properties of the HNF4α and the many interesting mechanisms through which signals impact this receptor to mediate their functions.

## Protein architecture and allostery

**Figure 1:**
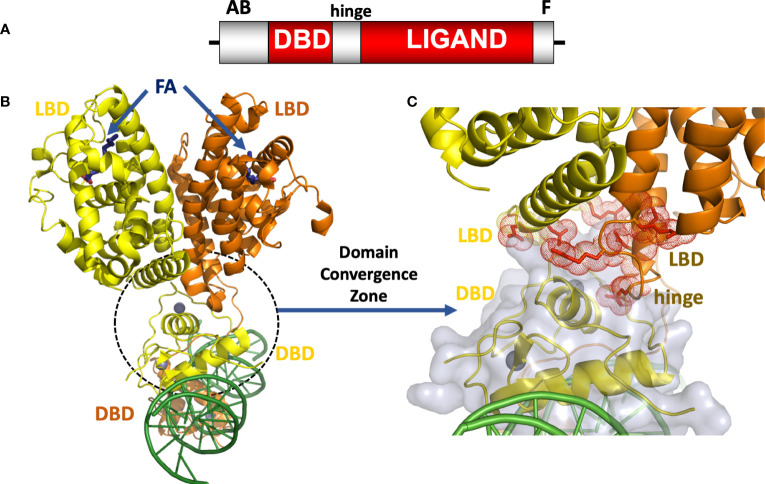
**(A)** The HNF4α domain arrangement. **(B)** Crystal structure of the HNF4α homodimeric complex bound to DR1 DNA with fatty acid (FA) ligands occupying the LBDs shown in blue, and zinc ions shown in grey (30), PDB 4IQR. **(C)** Close-up view of the domain convergence zone (circled) where amino-acid residues from LBDs, a DBD and a hinge segment converge to allow for allosteric communications between distal segments of the polypeptide.

Like all NRs, the HNF4α protein is comprised of discrete domains. **Figure 1A** shows the positions of the DNA binding domain (DBD), ligand binding domain (LBD), the hinge region, N-terminal A/B segment, and F-domain. The DBD is responsible for binding to specific DNA sequences in the response element. The LBD, on the other hand, is responsible for binding to small molecules, in this case fatty acids, which are required for the integrity and stability of that domain in HNF4α. In other NRs, ligand binding changes the LBD conformation to alter the binding affinity for coactivators or corepressors, but this has not been demonstrated for HNF4α, as coactivators appear to always bind in the presence of a bound fatty acid (27, 30–32). The dimerization ability that is often encoded in the LBD of NRs allows two NRs, in either homodimeric or heterodimeric form, to become functional transcription factor. HNF4α is an obligate homodimer, whereas other NRs can form heterodimers with the retinoid X receptor (RXR) as a common partner, or function as monomers (28, 33). In addition to the dimerization capability in the LBD, the DBD of NRs can cooperate to form a DNA-dependent dimerization interface which requires properly oriented and spaced DNA half-site motifs (28, 34–37).

The DBD of HNF4α, like those of other NRs, involves two zinc bound modules critical for producing an overall stable structure necessary for DNA-binding through direct repeat base-pair readout (30, 34, 35). Each zinc binding modules involves coordination of the metal by four cysteine residues that are absolutely conserved across the NR family. The LBD is composed of around twelve helices and several short beta strands, which together form a three-dimensional structure that can accommodate the binding of small molecules (27). The structure of the HNF4α LBD subunit was determined by X-ray crystallography by two independent groups, and these were among dozens of isolated NR LBD structures that were studied over a period spanning fifteen to twenty years (27). The structure of the isolated HNF4α DBD on a natural promoter has also been reported (38).

Insights about the multidomain organization in HNF4α came when my laboratory characterized it using X-ray crystallography. Throughout a decade of studies on NRs, we determined structures for PPARγ-RXRα heterodimer, HNF4α homodimer and RXRα-RARβ heterodimer all bound to DNA response elements, ligands, and coregulator peptides (30, 37, 39–41). Our goal was to reveal the more complete nature of NR polypeptides and their domain interdependence. These studies revealed at atomic detail the quaternary organizations of these receptor complexes on DNA, and pointed immediately to the unique sets of domain-domain interactions for each NR. Moreover, the advances allowed us to test our ideas as to how signals in one domain could be allosterically transmitted to distal receptor domains to modulate their respective functions, and how disease-causing mutations and post-translational modifications manifest their effects. Among the three complexes that we studied, the HNF4α homodimeric complex proved most revealing and insightful (30).

Before fully discussing the HNF4α protein’s allosteric landscape, it is useful to define allostery in clear terms. An allosteric system is one where a perturbation at a specific site causes a meaningful change in the conformation, dynamics or function of a distal site in the same protein. Concepts of protein allostery at the molecular level were first proposed by the Monod–Wyman–Changeux (MWC) and the Koshland–Némethy–Filmer (KNF) models (42, 43). Both models involve induced conformational changes. The distinct inter-changeable conformations in a protein can represent the active and inactive states, or more generally multiple conformational states based on the nature and location of the original signal.

Subsequent concepts of allostery have highlighted the contributions of dynamics, in which the structure does not necessarily change but the dynamics of the protein is changed, or alternatively the signal on one site shifts the protein conformational ensemble from one to another. It has been shown that individual domains of NRs such as the DBD that engages with a response element are sufficiently flexible and dynamic on their own to allow induced fit and reconfiguration when forming a complex on DNA (44, 45). However, this review will focus more on the classic interpretations provided by MWC and KNF in discussing allostery based on snapshots revealed by X-ray crystal structures. The dynamic nature of these events can be probed by NMR spectroscopy and Hydrogen-Deuterium Exchange Mass Spectrometry (H/D ExMS) approaches as shown for DNA-protein complexes or other NRs (30, 39, 46).

The intact HNF4α protein contains the domain arrangement shown in **Figure 1A**. All of our efforts to crystallize that full-length HNF4α were unsuccessful because the extreme ends of the proteins proved too flexible to be accommodated within an ordered crystal lattice. But by proteolytically probing its DNA-assembled homodimeric form, we could identify and subsequently crystallize a substantial segment corresponding to the DBD-hinge-LBD portions of the human HNF4α corresponding to residues 46-368 (30). The homodimeric complex of this polypeptide formed readily and bound to a direct-repeat DNA element with one base-pair spacing (DR1). For the purposes of crystallization, we used an idealized DR1 element, which is the most sensible and informative binding site for understanding the basis for the receptor’s overall DNA preference.

When further combined with coactivator derived peptides, the complex containing all the above-mentioned components could finally be crystallized, and its structure was solved and refined at 2.9 Å resolution. The HNF4α homodimer structure showed a surprising arrangement. While the LBD portions were arranged in a symmetrical fashion relative to each other, the overall complex when considering all the remaining parts, is asymmetrical because of the direct-repeat nature of the DR1 element and the requirement of the DBD portions to engage it in a head-to-tail fashion (**Figure 1B**). In comparing the relevant portions of the multi-domain HNF4α on the idealized DR1 with that of the isolated HNF4α DBD on a natural promoter, it becomes evident that nominal base-pair divergence away from consensus DR1 does not lead to significant changes in the DNA-binding interface, or likely impacts overall quaternary architecture including the DBD-DBD or DBD-LBD interfaces (38).

The structure of the HNF4α protein was multi-layered and multi-faceted, with structured motifs from both subunits converging to produce a highly cooperative system that was also tuned for detecting signals with great sensitivity (30). In closely examining the arrangement, we discovered a central convergence zone within the homodimer that simultaneously incorporated surfaces from both LBDs, the DBD of the upstream subunit, and the hinge region of the downstream subunit. This central convergence zone immediately presented as the communication hub for propagating signals between domains (**Figure 1C**). Furthermore, the LBD and DBD modules were physically aligned to work together in establishing the high-affinity DNA response element binding. When we measured the DNA binding affinity of the HNF4α DBD and hinge portion alone, we observed very weak binding to DR1. But when the LBD portion was included as in our crystallization construct, the receptor’s DNA affinity was enhanced 75-fold (30).

As for the contributions of the protein segments that were not in our crystallization construct, we did not detect further gains in DNA binding affinity when the N-terminal (AB region) or C-terminal (F region) portions of the polypeptide were included in the full-length polypeptide (30). The proteolytically sensitive nature of these N-terminal and C-terminal regions also suggested they were disordered and not able to form additional domain-domain interfaces. Instead, these portions likely contribute to the recognition of other proteins in the transcriptional complexes that are formed within cells. We again confirmed the ability of the protein to use fatty acids as ligands, as we observed clear electron density for a trapped fatty acid in the LBD, consistent in size and nature with myristic acid.

Since the HNF4α homodimer, PPARγ-RXRα and RXRα-RARβ complexes all were co-crystallized with the same DR1 element and also had coactivator derived peptides included, we have asked if these three NR quaternary architectures are related. Their shared DR1 element does help establish a similar DBD-DBD spacing in all complexes. However, that is where the similarities end. In the higher order quaternary arrangements of these complexes, the nature of domain-domain interactions is strikingly different. This also meant that each of these NR complexes presents different paths for signal propagation from one domain to another. As detailed below, we found that a number of reported disease associated mutations and post-translational modification sites locate to sensitive domain-domain interaction points of the HNF4α homodimer (30). These findings provided a working hypothesis that such modifications mechanistically impart their regulatory effects by relying on the domain-domain arrangements we observed.

## Fatty acid binding

The binding of ligands to most NRs occurs in an exchangeable fashion, allowing transcriptional activity to be altered from active to inactive states (29, 36). The ligand identification for HNF4α has been subject to some controversy. An initial report indicated that long-chain fatty acids in the form of acyl-CoA thioester forms could bind to the recombinant HNF4α ligand-binding domains based on ^14^C-labelled fatty acids. In particular, palmitoyl-CoA ((C16:0)-CoA) binding was demonstrated to have saturable binding with a *K*_d_ of 2.6 μM, in-line with its known concentration levels in liver cytosol (47). That report further indicated that binding to HNF4α was only possible for its acyl-CoA form, as the assays used indicated the same free fatty acid or the free CoA alone did not bind detectibly. Moreover, it was reported that these fatty acyl-CoAs enhanced the ability of the receptor to bind DNA, but had relatively small effects on transcriptional activation, as only a 2-fold activation level of transcription was observed. The study did not use a rigorous assessment of NR ligand based on ligand ability to alter the protein conformation or to recruit co-regulators to HNF4α.

**Figure 2:**
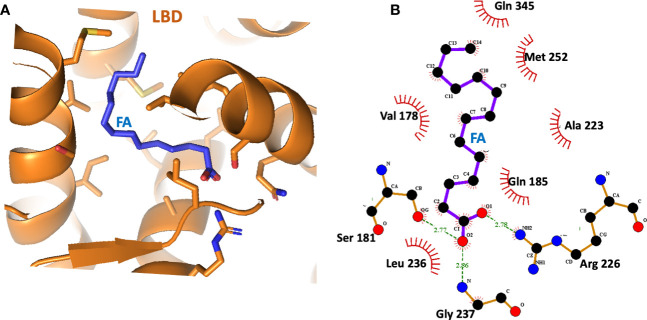
**(A)** The binding site of the fatty acid (myristic acid) inside the HNF4α LBD, derived from PDB 4IQR. **(B)** Close-up view of the amino-acid contacts formed between the LBD and the myristic acid. Red semi-circles indicate van der Waals contacts, and dotted lines show hydrogen-bonding interactions.

Subsequent studies on the ligand-binding properties of HNF4α showed there was no need for the acyl-CoA portion, instead it was found that the fatty acid chain length and saturation alone were the specificity elements required for direct binding (31, 32, 48). In separate crystal structures of the HNF4α ligand-binding domain (LBD) that were reported, a free fatty acid was found in the ligand binding site (31, 32). These studies again showed there was no acyl-CoA requirement for ligand binding. In our examination of the binding site of myristic acid within the LBD, we found the long hydrophobic segment of the fatty acid was totally encased within a hydrophobic cavity, whereas the hydrophilic carboxylic acid moiety pointed closer to the entrance of the pocket and hydrogen bonded to residues with polar side chains (**Figures 2A, B**). This form of encasing lipid molecules has been observable in other fatty acid-binding proteins that we have studied (49–52).

The crystallographic study on the LBD by Williams et al. further showed that HNF4α could bind directly and extremely tightly to saturated and cis-monounsaturated C14-18 fatty acids (31). These constituted a potential pool of fatty acids used by HNF4α as endogenous ligands, but the bound fatty acid did not readily exchange with radio-labelled palmitic acid, and all attempts to displace these ligands without denaturing the protein failed (31). Therefore, HNF4α transcription factor is believed to be constitutively bound by fatty acid acting not so much as a small-molecule sensor, rather the ligand plays an architectural role in folding and stability of the functional receptor.

Another study of HNF4α expressed in mammals showed it bound to the essential fatty acid linoleic acid (LA; C18:2), which was suggested to be a potential endogenous ligand of HNF4α (53). Although its binding may be reversible within cells, LA, like other free fatty acids did not appear to have any significant effect in modulating the transcriptional activation properties of HNF4α. LA could not transcriptionally activate HNF4α in mammalian cells exposed to lipid-depleted medium, even in the presence of PGC1α, a known coactivator of HNF4α (53).

## How disease mutations manifest

It is recognized that genetic and disease-causing mutations can act through allosteric sites (54–56). Mutations in the DNA-binding domain, ligand-binding domain, and other protein regions of HNF4α can each compromise overall transcriptional activity and lead to MODY1 (21). In addition, mutations in the DNA-binding sites of HNF4α, such as on the HNF-1α promoter, are also known to lead to MODY1 (57). Modifications of histone and DNA are two other mechanisms influencing DNA accessibility. Therefore, DNA methylation patterns of some binding sites may also alter gene expression, as found for HNF4α and other types of mammalian transcription factors (58, 59).

**Figure 3:**
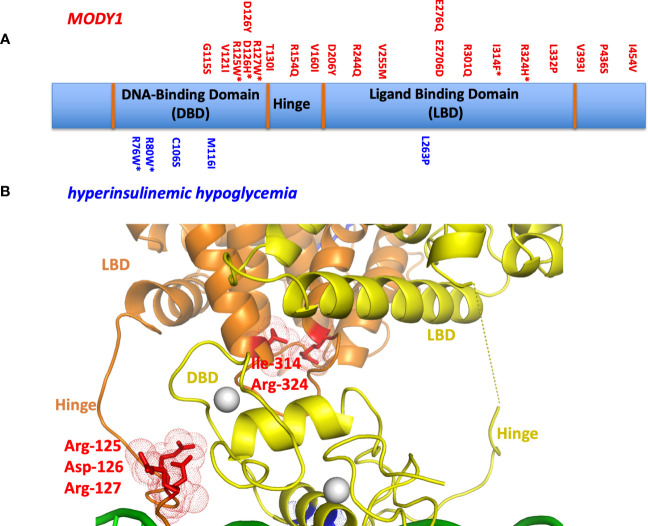
**(A)** The locations of MODY1 and HH mutations within the human HNF4α polypeptide. **(B)** Mapping of mutational sites on the three-dimensional structure (PDB 4IQR) shows many of these sites to be located within or in close proximity to the domain convergence zone.

One can study the locations of MODY1 and HH genetic lesions, since these are typically single point mutations, in the context of the crystallographic multi-domain HNF4α-DNA complex (**Figure 3A**). Their sites often lie at the most sensitive sites of the complex. Examples include MODY1 related mutations R127W, D126Y, D126H, and R125W. These all occur within the hinge portion of the receptor and could not be understood unless one could visualize the entire quaternary organization of the complex (**Figure 3B**). In the multi-domain setting, it became clear they all were located at the central communication hub/zone that was connecting the DBD and LBD and hinge region (30). These mutations in particular all cause the misalignment of the interaction between domain-domain surfaces and subsequently lead to compromised DNA binding in an allosteric fashion. In other words, their actions are located far away from their own actual sites on the linear polypeptide. These mutations also cause a reduction in the transcriptional activity of HNF4α.

We also examined MODY1 mutations I314F, R324H, and their adjacent residues (R322A, Q318A, D316A and N315A), which are all located relatively close to each other on the LBD (**Figure 3B**). Within the quaternary complex however, they also all physically map to the multi-domain convergence centre of the complex (30). These mutations also reduce the DNA affinity and transcriptional activity of the receptor allosterically. They are located on the LBD, but compromise the function of the DBD by manifesting their effects through a considerable distance across the linear polypeptide.

Other mutations had more simple explanations, in that they compromised a single functional site locally. For example, HH-associated R76W and R80W mutations were noted to compromise the DNA recognition helix as the arginine residues directly contact the AGGTCA half-sites from the DBD. Their substitutions with tryptophan would interfere with this base-pair readout. The MODY1 mutation V255M alters a residue inside the LBD pocket where the fatty acid is bound, disabling the binding of endogenous ligand(s) and potentially destabilizing the conformation of the LBD as a result (30).

## Posttranslational marks at allosteric sites

**Figure 4:**
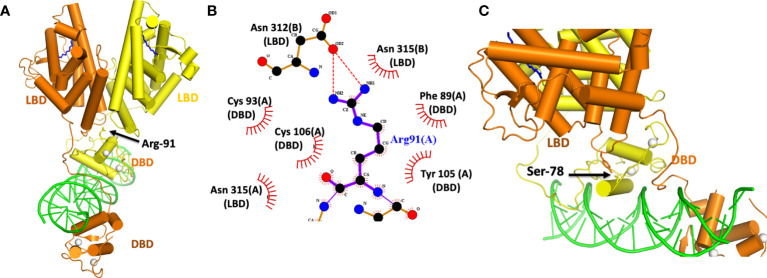
**(A)** Location of the Arg-91 targeted for methylation by PRMT1. **(B)** Close-up view of how Arg-91 from the DBD interacts with residues from the two LBDs. The A and B designations after each amino-acid number is consistent with two subunits in the homodimeric HNF4α. **(C)** Location of Ser 78 in the DBD targeted by PKC. This residue interacts with Tyr 315 from the LBD. Both Ser 78 and Tyr 315 fall inside the domain convergence zone, as seen in PDB 4IQR.

The activities of NRs can be subject to regulation by a variety of different post-translational modifications (PTMs). In the case of HNF4α, a pair of distinct PTMs were described to regulate the receptor’s function in gene expression. These sites have been examined within the HNF4α architecture to better understand their allosteric effects (**Figure 4**). The first site, Arg-91, is a target of PRMT1, an enzyme that dimethylates arginine side-chains (60). Arg-91 methylation enhances the DNA binding activity of HNF4α (60). The second site Ser-78, is targeted by protein kinase C (PKC) and weakens DNA-binding (61). Therefore, taken together, these two PTMs act in opposing ways to regulate HNF4α activity.

Interestingly, while Arg-91 methylation enhances DNA-affinity, it was not located in a region of DBD that contacts DNA (**Figures 4A, B**). Instead, its side chain inserts physically into the allosteric convergence zone (**Figure 4A**). A cavity for this arginine side chain exists to accept the two extra methyl groups that are added by PRMT1 enzyme, and the arginine methylation and insertion cements the integrity of that convergence zone. Because this step stabilizes the overall quaternary structure, it can lead to a higher DNA-affinity as the LBDs are now better reinforced to support the DBD.

PKC targets HNF4α and also a number of other NRs (61). These other targets include FXR, RAR, VDR, PPARα, PXR and TR2, and importantly the phosphorylated serine is always positioned similarly along the DBD. The location, however, is on “wrong side” of the DNA recognition helix that contacts the DNA (**Figure 4C**). Yet Ser-78 phosphorylation still weakens receptor-DNA binding (61). But in the quaternary arrangement of the HNF4α complex, Ser-78 is located at domain-domain junctions, and therefore its ability to weaken DNA binding occurs through allostery (**Figure 4C**). A PKC introduced phosphate group on this serine is expected to cause unfavourable interactions between Ser-789 and Tyr-319, weakening the receptor LBD to DBD connections that otherwise formed through their physical interactions.

The careful analysis of the positional effects of PTMs, MODY1 and HH mutations point clearly to allostery in this protein-DNA complex. Perturbations within the LBD, the hinge region, or in the DBD are transmitted across the complex to distal sites. It is remarkable that the subtlety of a single PTM or a single amino-acid change can be transmitted across such large distance of the polypeptide efficiently. The domain convergence zone of HNF4α is indeed a highly sensitive centre for receiving signals and for propagating signals in a functionally meaningful way.

## Discussion

It is highly likely that other classes of transcription factors, particularly those that utilize homo- and heterodimerization and have multiple domains, also transmit information between their domains allosterically to modulate their functions. Of course, allostery was first noted in the bacterial lac repressor (42). But for mammalian transcription factors, this has been more difficult to illuminate due to the lack of information about their multi-domain structural complexes. One class of mammalian transcription factors, known as basic-loop-helix PER-ARNT-SIM (bHLH-PAS) family, are increasingly becoming structurally elucidated for their multi-domain architectures, and the allosteric effects of their ligands. This group of transcription factors also harbour internal pockets that can be used for ligand binding. As a result, the bHLH-PAS proteins are increasingly seeing the discovery of novel and highly potent agonists and antagonists, the identification of endogenous ligands, and potential paths to the clinic for newly identified drugs (49, 62–69).

Nevertheless, the nuclear receptors still stand as the most successful class of transcription factors for having approved drugs that bind directly to their polypeptides (70). This success stems largely from the hydrophobic pockets offered by their LBDs which normally serve the purpose of binding to endogenous ligands. Importantly, HNF4α has not seen a drug approved to date, largely because the fatty acid interactions with its LBD are high-affinity and difficult to displace. The lessons learned to date about HNF4α protein architecture and domain-domain interfaces have elevated the overall appreciation for thinking outside the LBD fold when considering how ligands act through these receptors. In the case of the RXRα-RARβ heterodimer, studies have shown that LBDs and DBDs can sense and transmit their local information to distal elements within the complex in an allosteric manner (37).

Analysis of our three multi-domain co-crystal structures (PPARγ-RXRα heterodimer, HNF4α homodimer and RXRα-RARβ heterodimer) by X-ray crystallography, and subsequent NR complex structure determinations consistently find domain-domain contacts and physical interactions within all multi-domain complexes (30, 37, 39, 40, 71). These are consistently seen as closed conformations with clear-cut domain-domain contacts. Evidence for their domain-domain contacts has also emerged from solution studies employing H/D ExMS and small-angle X-ray scattering studies (30, 37, 39, 72). The closed conformations detected by these methods are found to match the crystallographically observed domain-domain interactions very closely.

My laboratory and others have pointed out that some other low-resolution structural models for multi-domain nuclear receptor complexes on direct-repeats, such as those generated by the Dino Moras lab and his colleagues, are not supported by any crystallographic, H/D ExMS, or mutagenesis studies. Those so-called open conformations also could not explain known lipodystrophy mutations in PPARγ, where the PPARγ-RXRα crystallographic complex provide an excellent explanation based on the observed DBD-LBD interactions of the closed structure (37, 40, 73).

The role of endogenous fatty acid ligands for HNF4α (60) still presents an important gap in our understanding of this receptor’s function and regulation. If the fatty acids are not exchangeable and their role is to stabilise the protein fold only, then they are unlikely to act as functional modulators in the way other NR ligands are understood to do. The regulation of the receptor instead may be largely derived from PTMs. The “natural” state of HNF4α may have a fairly high level of constitutive activity since the fatty acids produce a strong stabilizing effect on the folding of the active conformation. It would be helpful to develop new tools and methodologies to detect fatty acid binding and measure the associated transcriptional activities that they impart, since the cellular environment presents significant challenges to these explorations. To test the actual extent of fatty acid contribution to protein stability, one could measure protein stability either intracellularly or biochemically. In a biochemical experiment, stability assessment could be conducted using a protein thermal shift assay, whereas in a cellular experiment the protein half-life and turnover could prove useful indicators.

In mammalian cells, any experiments with individual fatty acids are certainly challenging to configure. A good case for linoleic acid as an endogenous ligand has been made to date, while also recognizing that this molecule may have little effect on transcriptional activity (53). It is unclear if the ligand-free state of HNF4α even exists within cells, so it is not clear what the off-state of the protein entails in terms of transcriptional activation or repression. One creative way to access the unliganded state at the cellular level, is to rely on the point mutation V255M that falls within its binding pocket. Other mutational changes that disable fatty acid binding could be introduced stably and genetically into mice, and tissue based genomic profiling and whole animal physiology could be studied to assess what is lost or gained *versus* wild-type mice. Intracellularly, it would be interesting to identify which lipids in pancreatic β-cells bind to the HNF4α LBD and whether these are distinct from linoleic acid that appears to be the candidate ligand from the liver.

There is also more to learn about the structure of HNF4α, particularly the contributions of the F-domain and the structure and functional consequences of its many known isoforms. Indeed, in this context, more than 60 potential isoforms exist which may show differential gene expression or regulation (74, 75). In addition to alternative splicing, HNF4α isoforms are generated by two alternative promoters, P1 and P2, giving rise to proteins that can include or lack an additional N-terminal transactivation domain (76–79). HNF4α isoforms have different abilities to interact with DNA and transcriptional cofactors, and a clear understanding of these variations based on a structural framework remains unavailable (74, 80).

While HNF4α has mainly been described within this review in the context of a transcriptional activator promoting recruitment of coactivators, some studies have also indicated that HNF4α can interact with corepressors in an F-domain dependent or isoform specific manner to suppress gene expression (81, 82). Even on a given gene, different HNF4α isoforms can function as a transcriptional activator or repressor (74). In all these areas, a mechanistic or structural understanding related to versatility of HNF4α to both positively or negatively impact gene expression in isoform dependent manner is well worth exploring.

## Author contributions

The author confirms being the sole contributor of this work and has approved it for publication.
